# Evaluation and Comparison of Contemporary Energy-Based Surgical Vessel Sealing Devices

**DOI:** 10.1089/end.2017.0596

**Published:** 2018-04-01

**Authors:** Zhamshid Okhunov, Renai Yoon, Achim Lusch, Kyle Spradling, Melissa Suarez, Kamaljot S. Kaler, Roshan Patel, Christina Hwang, Kathy Osann, Jiaoti Huang, Thomas Lee, Jaime Landman

**Affiliations:** ^1^ Department of Urology, University of California, Irvine, Orange, California.; ^2^ Department of Urology, University of California, Irvine, Orange, California.; ^3^ Department of Pathology, Duke University, Durham, North Carolina.; ^4^ Department of Pathology, University of California, Irvine, Orange, California.

**Keywords:** vessel sealing technologies, laparoscopic hemostasis, surgical energy devices, LigaSure, Harmonic Scalpel, EnSeal, Caiman

## Abstract

***Introduction:*** We evaluated and compared five currently available energy-based vessel sealing devices to assess typical surgical metrics.

***Methods:*** We tested Caiman 5 (C5), Harmonic Scalpel Ace Plus (HA), Harmonic Ace +7 (HA7), LigaSure (LS), and Enseal G2 (ES) on small (2–5 mm), medium (5.1–7 mm), and large (7.1–9 mm) vessels obtained from 15 Yorkshire pigs. Vessels were randomly sealed and transected. We recorded sealing and transection time, charring and carbonization, thermal spread, and bursting pressure (BP). Specimens were sent for histopathologic evaluation of seal quality and thermal spread.

***Results:*** A total of 246 vessels were evaluated: 125 were arteries and 121 were veins. There was no difference in BPs for small size arteries. For medium arteries, C5 provided the highest BP (proximal and distal jaw), followed by HA7, ES, LS, and HA [1740, 1600, 1165, 1165, 981, and 571 mm Hg, respectively, HA<C5-D(<0.001); HA<C5-P(<0.001); HA<ES(0.002); HA<HA7(0.002); HA7<C5-P(0.026); ES<C5-P(0.026); LS<C5-P(0.001); LS<C5-D(0.014)]. For large arteries, C5 and LS provided highest BP followed by HA7, ES, and HA [1676, 530, 467, 467, and 254 mm Hg, respectively, C5<HA(<0.001); C5<HA7(0.006); C5<ES(0.006); C5<LS(0.012)]. There were no bursting pressure failures for C5, HA7, and LS up to 9 mm vessels. For medium and large size arteries, HA had bursting failure of 20% and 40%, respectively. The ES was significantly less efficient with small, medium, and large arteries with bursting failure rates of 10%, 40%, and 80%, respectively.

***Conclusions:*** In this study, C5 outperformed all other devices. However, all of the devices provide a seal that was superphysiologic in that all burst pressures were >250 mm Hg.

## Introduction

Energy-based vessel sealing devices (VSDs) have been developed to facilitate dissection and hemostasis during open and laparoscopic procedures.^1^ These technologies enable surgeons to improve the efficiency and safety of procedures with decreased blood loss and operative times.^2^ In contemporary practice, there is continuous expectation for technological improvement to produce high-quality VSDs with more precise vessel sealing quality and reduced thermal injury to surrounding tissues.^1^

Traditional monopolar devices are associated with unpredictable and weak vessel sealing and increased lateral thermal spread.^3,4^ Bipolar energy and ultrasonic devices are the most routinely used hemostatic energy devices in contemporary surgical practice. Contemporary bipolar devices utilize electrothermal energy to seal blood vessels and reduce damage to surrounding tissues by preventing electric current from spreading beyond the jaws of the device. In comparison, ultrasonic devices utilize high-frequency vibrations instead of electrical current but the energy is again limited to being delivered just between the jaws of the device with scant lateral spread.

The reliability of vessel sealing using energy devices is clinically vital; in this regard, it is important for the seal quality to be consistent. Continuous research and technological advancements have significantly improved contemporary VSDs. New design improvements have been developed to provide more consistent energy delivery and a feedback loop to optimize seal quality.

We evaluated and compared five contemporary commercially available energy-based VSDs in an *in vivo* animal model with regard to mesenteric transection time, vessel transection time, vessel sealing time, jaw temperature, thermal spread, and seal burst pressures.

## Methods

### Study design

We obtained an Institutional Animal Care and Use Committee approval to perform the study experiments. We evaluated five VSDs: the Caiman 5 (C5; Aesculap, Inc., Center Valley, PA), Harmonic Scalpel Ace Plus (HA; Ethicon Endosurgery, Cincinnati, OH), Harmonic Ace +7 (HA7; Ethicon Endosurgery), LigaSure (LS; Covidien, Mansfield, MA), and Enseal G2 (ES; Ethicon Endosurgery). We used each VSD according to the settings recommended by the manufacturer.

There were three phases to the study methodology, including *in vivo* dissection, *ex vivo* testing of bursting pressure, and finally histopathologic examination of vessel seals.

We used 15 Yorkshire pigs (30–35 kg) to evaluate the 5 devices (total 3 pigs per device). The surgeons performing the testing (Z.O. and J.L.) were experienced with all the energy devices. Despite surgeon familiarity, one pig was utilized to train the surgeons on tissue and vessel sealing in the porcine model to mitigate any device-specific learning curve.

### Tissue transection

After adequate general endotracheal anesthesia, a midline incision was made from xiphoid process to symphysis pubis. Once exposed, we inked the colonic mesentery at 15-cm increments and performed a timed mesenteric transection. Mesentery transection time was defined as the time needed to dissect a 15 cm segment of small bowel mesentery. For each VSD, we performed a total of 10 transections 15 cm in length. Devices were cleaned between each transection to maintain device performance. The surgeon rated charring/carbonization, tissue sticking, seal quality, and transection quality on a 1–5 scale (1 best and 5 worst, Table 1) based on visual estimation of the seal for both the upper and lower jaws of each VSD. We measured and recorded the maximum jaw temperature for each VSD using a thermal camera (FLIR E5; FLIR Systems, Boston, MA).

**Table 1.** Scoring Criteria and Definitions

	*Definition*
Score	Charring/carbonization
1	No charring/carbonization
2	Slight charring/carbonization that does not interfere with sealing/transection
3	Slight charring/carbonization on upper or lower jaw requiring cleaning
4	Moderate charring/carbonization on one or both jaws requiring cleaning
5	Significant charring/carbonization on both jaws requiring cleaning
Score	Tissue sticking
1	No tissue sticking
2	Slight sticking requiring activation of the device to release tissue
3	Tissue sticking requiring counter tension to gently remove tissue
4	Tissue sticking requiring counter tension and extensive force to remove tissue
5	Tissue sticking such that tissue is damaged or torn during the removal process
Score	Seal quality
1	Excellent, no bleeding
2	Blood oozing at tissue site
3	Blood oozing at tissue site at 5 seconds
4	Moderate bleeding requiring intervention
5	Bleeding without evidence of tissue sealing
Score	Transection quality
1	Complete tissue transection from proximal to distal end of the jaw
2	Incomplete tissue transection cut proximally, not distally
3	Incomplete tissue transection cut distally, not proximally
4	Incomplete tissue transection with bleeding
5	No tissue transection occurred

### Vessel sealing and transection

The aorta and vena cava were exposed. The renal arteries and renal veins and their branches were cleared of all surrounding tissue as were the iliac and femoral arteries and veins. The dissection was continued down to the smallest macroscopic peripheral vessels. In gaining access to the femoral arteries, we extended the inferior midline incision onto the medial side of hind legs bilaterally. In addition, we exposed carotid arteries and veins through a ventral cervical incision with further clearing of surrounding tissue to expose these vessels and their branches. We measured the diameter of each vessel with digital calipers before sealing and transection. We then divided the vessels into three categories: small (2–5 mm), medium (5.1–7 mm), and large (7.1–9 mm). Testing on each VSD was performed on each of these three categories. The C5 instrument has a special jaw-closing mechanism with an additional hinge at approximately half of the length of the lower jaw part. This results in even pressure distribution and tissue compression within the jaws and enables an increased jaw length in comparison with scissor-like jaw mechanisms. As such, for C5, we tested sealing and transection properties in the proximal and distal jaws separately for small and medium vessels. For large vessels, testing was performed in the middle component of C5 only as the vessels were too large to fit in the separate components. We evaluated sealing and transection of vessels with all other devices, with the vessels secured in the middle of the jaw. Once sealed and transected, each VSD and vessel was visually rated by the surgeon for charring/carbonization, tissue sticking (1 best to 5 worst), and seal quality (1 best to 5 worst) using standardized scoring criteria (Table 1). In additional, we measured the maximum jaw temperature for each VSD using a thermal camera (FLIR E5; FLIR Systems).

### Bursting pressure

After *in vivo* sealing, we performed the bursting pressure in an *ex vivo* setting as described previously.^3^ We inserted an 18-gauge angiocatheter into the lumen of each vessel tested. We allowed at least 1 cm vessel length to provide enough space between the needle tip and the vessel seal. Once inserted, we secured the angiocatheter to the vessel with a silk suture and a hemostat clamp. The angiocatheter was then attached to both a digital pressure manometer (Deluxe Digital Manometer DM8200; General Tools, New York, NY) and a saline pump with tubing. Physiologic saline was instilled at a steady rate until leakage or vessel bursting was observed on the sealed section of the vessel. Bursting pressure was recorded in mm Hg.

### Bursting pressure failures

We defined the arterial bursting failure as vessel bursting less than a pressure of 300 mm Hg. We defined the venous bursting pressure failure as vessel seal bursting at a pressure <30 mm Hg.^5–7^

### Histopathology

For histopathologic evaluation of thermal spread and seal quality, we collected treated vessels that had not been burst tested after sealing. These vessels were placed into 10% neutral buffered formalin and subsequently were embedded in paraffin wax and sectioned. These segments were then evaluated by an expert pathologist for thermal damage and seal quality (hematoxylin and eosin staining). The pathologist was blinded as to which VSD was used. The extent of thermal energy damage was defined by measuring the length of coagulation necrosis from the seal.

### Statistical analysis

We used analysis of variance (*F*-test, Kruskal–Wallis test) to test for the differences among the groups; each device served as an independent grouping factor. Differences between distal and proximal vessel sealing were explored by adding an additional grouping factor. We used SYSTAT statistical software, version 13.0, to analyze the data (Systat Software, Inc., San Jose, CA). Statistical significance was defined as *p*-values <0.05.

## Results

A total of 246 vessel segments including carotid, renal, iliac, and femoral vessels were obtained from 15 animals. All vessels were sealed and transected using one of the VSDs. Arteries and veins were equally distributed between different VSDs according to size criteria used for bursting pressure tests. Out of the 246 vessels, 25 arteries and veins were sent directly for histopathologic evaluation of thermal spread and seal quality.

### Tissue transection

A total of fifty 15-cm mesentery segments were transected: 10 segments for each VSD. Transection time, charring and carbonization, tissue sticking, and maximum jaw temperature measurements are provided in Table 2.

**Table 2.** Comparison of Tissue Transection Time, Charring and Carbonization, Tissue Sticking, and Maximum Jaw Temperature Measurements Between Caiman 5, Enseal G2, Harmonic Ace Plus, Harmonic Ace +7, and LigaSure

*Variables*	*C5*	*ES*	*HA*	*HA7*	*LS*	p	*Pairwise* t*-tests (Tukey adjustment for multiple comparisons)*
Transection time, mean (SD)	67.3 (3.9)	96.5 (3.9)	61.6 (3.9)	60.8 (3.9)	91.1 (3.9)	<**0.001**^a^	ES>C5(<0.001); ES>HA(<0.001); ES>HA7(<0.001); LS>C5(0.001); LS>HA(<0.001): LS>HA7(<0.001)
Charring/carbonization—up, median (range)	1 (1–1)	1 (1–2)	1 (1–2)	1 (1–1)	1 (1–3)	0.429^b^	No pairwise differences are significant
Charring/carbonization—low, median (range)	1 (1–1)	1 (1–1)	1.5 (1–3)	1 (1–1)	1 (1–3)	**<0.001**^b^	HA>C5(0.001); HA>ES(0.001); HA>HA7(0.001); HA>LS(0.001)
Tissue sticking, median (range)	1 (1–1)	1 (1–1)	1 (1–3)	1 (1–3)	4 (3–5)	**<0.001**^b^	LS>C5(<0.001); LS>ES(<0.001); LS>HA(<0.001); LS>HA7(<0.001)
Transection quality, median (range)	1 (1–1)	1 (1–1)	1 (1–1)	1.2 (1–2)	1.2 (1–2)	0.170^b^	No pairwise differences are significant
Maximum jaw temperature, mean (SD)	109 (3.4)	114 (3.4)	195.6 (3.4)	260.1 (3.4)	95 (3.4)	**<0.001**^a^	HA7>C5(<0.001); HA7>ES(<0.001); HA7>HA(<0.001); HA7>LS(<0.001); HA>C5(<0.001); HA>ES(<0.001): HA>LS(<0.001); C5>LS(0.043); ES>LS(0.002)

^a^ *F*-test.

^b^ Kruskal–Wallis test.

All significant *p*-values are at *p* < 0.05.

C5 = Caiman 5; ES = Enseal G2; HA = Harmonic Ace Plus; HA7 = Harmonic Ace +7; LS = LigaSure.

HA7 demonstrated the fastest mesenteric transection time with the mean of 60.8 seconds, followed by HA, C5, LS, and ES demonstrating 61.5, 67.3, 91, and 96.5 seconds, respectively [ES>C5(<0.001); ES>HA(<0.001); ES>HA7(<0.001); LS>C5(0.001); LS>HA(<0.001); LS>HA7(<0.001)]. There were no differences in the median charring/carbonization scores (1.0, 1.1, 1.3, 1.1, and 1.0 for C5, HA, LS, ES, and HA7, respectively, no pairwise differences were significant). The median tissue sticking scores were 1.0, 1.2, 1.6, 1.0, and 4.2 for C5, HA, HA7, ES, and LS, respectively [(LS>C5 (<0.001); LS>ES(<0.001); LS>HA(<0.001); LS>HA7(<0.001)]. Transection quality was equal among all VSDs: 1 for C5, 1 for HA, 1.2 for HA7, 1.2 for LS, and 1.0 for ES (no pairwise differences were significant). Maximum jaw temperature recorded during the mesenteric transections was highest for HA7 followed by HA, ES, C5, and LS (260°C, 114°C, 109°C, 95.6°C, and 95°C, respectively, [HA7>C5 (<0.001); HA7>ES(<0.001); HA7>HA(<0.001); HA7>LS(<0.001); HA>C5(<0.001); HA>ES(<0.001); HA>LS(<0.001); C5>LS(0.04); ES>LS(0.002)].

### Vessel sealing and transection, and bursting pressures

Mean vessel sizes, percentage of *in vivo* failure, mean vessel bursting pressures, and mean maximum jaw temperature for arteries and veins comparing all VSDs are given in Tables 3 and 4.

**Table 3.** Arteries

	*C5 distal*	*C5 proximal*	*HA*	*HA7*	*LS*	*ES*	p	p*-Value for pairwise comparisons (Tukey adjustment for multiple comparisons)*
Small (2–5 mm)								
*N*	5	5	10	10	10	10		
Vessel size, mm (mean)	3.6	2.8	2.9	2.4	3.1	2.9	0.153	None significant
Bursting pressure, mm Hg (mean)	1580	1405	1189	1506	1311	1506	0.273	None significant
Maximum jaw temperature	130	126	162	171	81.5	98	<**0.001**^a^	LS<HA(0.002); LS<HA7(0.002); ES<HA(0.017); ES<HA7(0.015)
Percentage of burst pressure failure	0	0	0	0	0	10	**<0.001**^a^	
Medium (5.1–7 mm)								
*N*	5	5	10	10	10	10		
Vessel size, mm (mean)	5.6	5.2	5.7	5.8	5.9	6.1	0.09	None significant
Bursting pressure, mm Hg (mean)	1600	1740	571	1165	981	1165	**<0.001**^a^	HA<C5-D(<0.001); HA<C5-P(<0.001); HA<ES(0.002); HA<HA7(0.002); HA7<C5-P(0.026); ES<C5-P(0.026); LS<C5-P(0.001); LS<C5-D(0.014)
Maximum jaw temperature	120.6	123.4	151.8	130.4	87	111.7	**<0.001**^a^	LS<C5D(<0.001); LS<C5P(<0.001); LS<HA(<0.001); LS<HA7(<0.001); LS<ES(0.001); ES<HA(<0.001); ES<HA7(0.023); C5D<HA(0.001); C5P<HA(0.002); HA7<HA(0.006)
Percentage of burst pressure failure	0	0	20	0	0	40	**<0.001**^a^	
Large (7.1–9 mm)								
*N*		5	5	5	5	4		
Vessel size, mm (mean)		7.6	7.7	7.8	7.2	7.4	0.756	None significant
Bursting pressure, mm Hg (mean)		1676	254	467	530	467	**<0.001**^a^	C5<HA(<0.001); C5<HA7(0.006); C5<ES(0.006); C5<LS(0.012)
Maximum jaw temperature		125	213	156	91.3	121	**<0.001**^a^	C5<HA(0.001); HA7<HA(0.014); LS<HA(<0.001); ES<HA(<0.001); LS<HA7(0.006)
Percentage of burst pressure failure		0	40	0	0	80	**<0.001**^a^	

^a^ Significant at *p* < 0.05.

**Table 4.** Veins

	*C5 distal*	*C5 proximal*	*HA*	*HA7*	*LS*	*ES*	p	p*-Value for pairwise comparisons (Tukey adjustment for multiple comparisons)*
Small (2–5 mm)								
*N*	5	5	10	10	10	10		
Vessel size, mm (mean)	3.0	3.1	2.9	2.8	3.3	2.8	0.647	None significant
Bursting pressure, mm Hg (mean)	743	1048	447	533	730	533	0.009^a^	HA<C5P(0.02); HA7<C5P(0.001); ES<C5P(0.001)
Maximum jaw temperature	128	125	162	164	90	100	0.022^a^	LS<HA7(0.001); LS<HA(0.012); ES<HA7(0.001)
Percentage of burst pressure failure	0	0	0	0	0	0	n/a	n/a
Medium (5.1–7 mm)								
*N*	5	5	10	10	10	10		
Vessel size, mm (mean)	5.9	5.4	5.9	5.7	5.9	5.7	0.554	None significant
Bursting pressure, mm Hg (mean)	704	730	271	560	464	560	<0.001^a^	HA<C5P(0.022); HA<C5D(0.017; LS<C5P(0.010); LS<C5D(0.018)
Maximum jaw temperature	126	123	170	184	89	104	<0.001^a^	LS<HA7(0.001); LS<HA(0.001); ES<HA7(0.001); ES<HA(0.001); C5P<HA7(0.001)
Percentage of burst pressure failure	0	0	0	0	0	0	n/a	n/a
Large (7.1–9 mm)								
*N*		5	5	5	5	5		
Vessel size, mm (mean)		8.9	8.6	8.6	8.2	8.6	0.833	None significant
Bursting pressure, mm Hg (mean)		449	336	446	364	446	0.877	None significant
Maximum jaw temperature		123	193	186	96	118	<0.001^a^	LS<HA7(0.021); LS<HA(0.001); ES<HA7(0.001); ES<HA(0.001); C5<HA7(0.001); C5<HA(0.012)
Percentage of burst pressure failure		0	0	0	0	0	n/a	n/a

^a^ Significant at *p* < 0.05.

### Seal quality, tissue sticking, and charring/carbonization

#### Arteries

For small arteries, seal quality scores were equal for distal and proximal C5, HA, HA7, and LS, which were superior to ES [1.2, 1.0, 1.0, 1.1, 1.2, and 2.3, respectively, C5P<ES(0.017); HA<ES(0.002); HA7<ES(0.005); LS<ES(0.012)]. For medium size arteries, seal quality scores for distal and proximal C5, HA, HA7, LS, and ES were 1.0, 1.0, 1.0, 2.8, 1.6, and 1.4, respectively, [C5D<HA(0.046); C5P<HA7(0.046); HA<HA7(0.008)]. For large size arteries, the mean seal quality scores were 1.3, 1.0, 3.7, 1.0, and 1.0 for C5, HA, HA7, LS, and ES, respectively (*p* = 0.046; no pairwise differences were significant).

Tissue sticking scores for small arteries were significantly better for distal and proximal C5, HA, and HA7 than for LS and ES [1.2, 1.4, 1.3, 1.5, and 3.5, 3.3, respectively, *p* < 0.001; C5P<LS(0.026); HA<LS(0.002); HA7<LS(0.006); HA<ES(0.006); HA7<ES(0.017)]. Tissue sticking scores for medium size arteries were better for distal and proximal C5 followed by HA, ES, HA7, and LS [1.2, 1.2, 1.8, 2.8, 3.6, and 3.2, respectively, *p* = 0.001; C5D<HA7(<0.001); C5D<LS(0.003); C5D<ES(0.029); C5P<HA7(<0.001); C5P<LS(0.003); C5P<ES(0.029); HA<HA7(0.001); HA<LS(0.016)]. Tissue sticking for large arteries was superior for HA7 and ES followed by C5, LS, and HA [1.0, 1.0, 1.7, 1.7, and 3.3, respectively, *p* = 0.006; ES<HA(0.008); HA7<HA(0.008)].

The LS resulted in significantly more charring and carbonization than the other devices. Specifically, charring and carbonization for small arteries were 1.4, 1.6, 1.1, 1.4, 2.8, and 1.3 for distal and proximal C5, HA, HA7, LS, and ES, respectively [*p* = 0.004; ES<LS(0.011); HA<LS(0.003); HA7<LS(0.022)]. Charring and carbonization for medium size arteries were 1.2, 1.2, 1.3, 1.0, 4.5, and 2.3 for distal and proximal C5, HA, HA7, LS, and ES, respectively [*p* < 0.001; C5D<LS(<0.001); C5P<LS(<0.001); HA<LS(<0.001); HA7<LS(<0.001); ES<LS(0.005)]. Charring and carbonization for large arteries were 2.3, 2.3, 1.0, 2.3, and 1.0 for distal and proximal C5, HA, HA7, LS, and ES, respectively (*p* = 0.39; no pairwise differences are significant).

#### Veins

For small size veins, seal quality scores were better for distal and proximal C5, LS and inferior for HA, HA7, and ES [1.0, 1.0, 1.0, 1.4, 1.4, and 2.4, respectively (*p* = 0.003; C5P<ES(0.001); C5D<ES (<0.001), HA<ES(0.024); HA7<ES(<0.001); LS<ES(0.016)]. For medium size veins, seal quality scores were better for distal and proximal C5, HA, and LS and inferior for HA7 and ES [1.0, 1.0, 1.0, 1.4, 2.8, and 3.2, respectively, *p* = 0.017; C5P<ES(0.021); HA<ES(0.034); HA7<ES(0.01); LS<ES(.012); C5P<HA7(0.001); HA<HA7(0.003); LS<HA7 (0.001)]. For large size veins, C5 and LS demonstrated significantly better seal quality followed by HA, ES, and HA7 (1.8, 1.8, 2.8, 3.2, and 3.8, respectively, *p* = 0.026; no pairwise differences were significant).

Tissue sticking scores for small veins were significantly better for distal and proximal C5, HA, and HA7 and inferior for LS and ES [1.1, 1.2, 1.2, 1.6, 3.6, and 3.4, respectively, *p* = 0.020; C5P<LS (0.002); C5D<LS(<0.001); HA<LS(0.031); HA7<LS(0.01); C5P<ES(0.02); C5D<ES(<0.001); HA<ES(0.046); HA7<ES(0.01)]. Tissue sticking scores for medium size veins were better for distal and proximal C5 and ES followed by HA, HA7, and LS [1.4, 1.6, 1.8, 2.4, 3.4, and 3.6, respectively, *p* = 0.001; C5P<LS(<0.001); C5D<HA7(<0.01)]. Tissue sticking for large veins was superior for C5, HA7, and ES followed by HA and LS (1.7, 2.0, 2.0, 3.5, and 3.7, respectively, *p* = 0.006; no pairwise differences were significant).

Charring and carbonization for small veins were significantly better for distal and proximal C5, HA, HA7, and ES and inferior for LS [1.2, 1.6, 2.1, 1.3, 2.7, and 1.3, respectively, *p* = 0.024; ES<LS(0.033)]. Charring and carbonization for medium size veins for distal and proximal C5, HA, HA7, LS, and ES were 1.8, 1.4, 1.3, 1.0, 4.2, and 2.2 [*p* = 0.001; C5D<LS(0.015); C5P<LS(0.003);HA<LS(<0.001); HA7<LS(<0.001); ES<LS(0.012)]. Charring and carbonization for large size veins for distal and proximal C5, HA, HA7, LS, and ES were 1.7, 2.3, 1.0, 2.3, and 1.3 (*p* = 0.593; no pairwise differences were significant).

### Seal quality and thermal spread

**FIG. 1.**
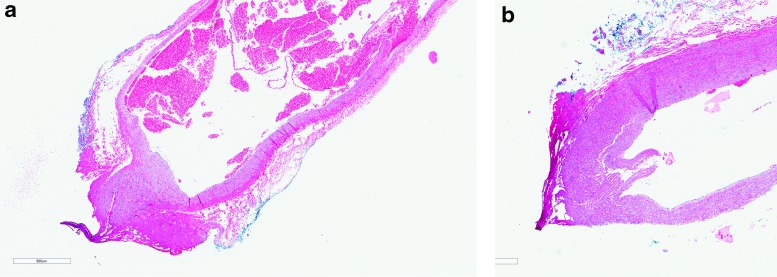
**(a)** Seal quality and thermal spread on a vein using Harmonic Ace +7—section of artery with soft tissue cautery changes consisting of smudging of the vessel wall morphology (dense acellular eosinophilia; *left lower corner*); however, cellular details of the smooth muscle within the central portion of the cautery edge are still fairly well preserved. **(b)** Seal quality and thermal spread on an artery using Harmonic Ace +7—section of artery with mild cautery changes as described in Fig. 1a, predominantly in the periphery of the tissue edge (*left side*), with good preservation of the greater portion of the cellular details of the vessel wall immediately beneath the cautery changes.

**FIG. 2.**
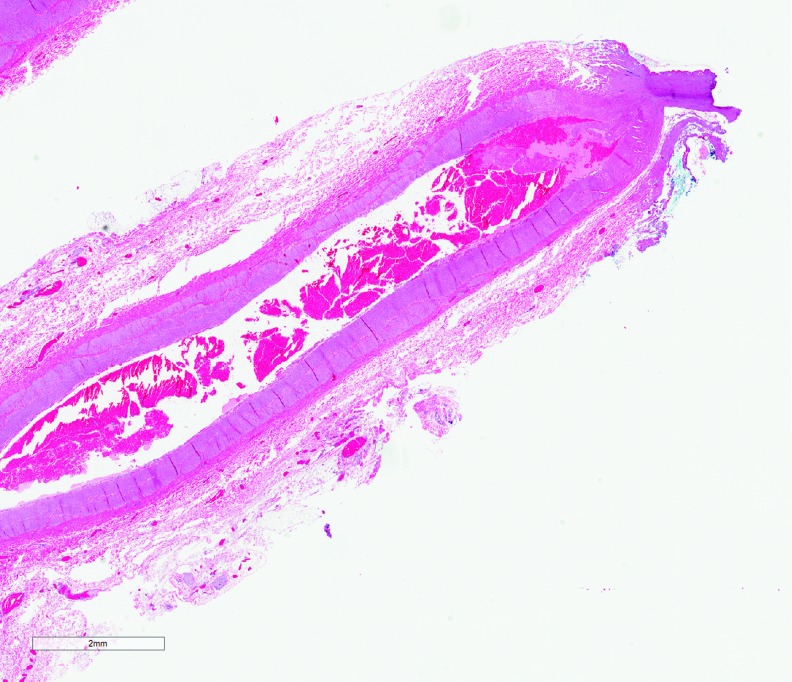
Seal quality and thermal spread on an artery using Caiman 5—section of artery with moderate to severe cautery changes seen in the vessel wall with adherent fibrin and moderate loss of cellular detail of the vessel wall (*upper right*). The cautery changes permeate into the vessel wall.

**FIG. 3.**
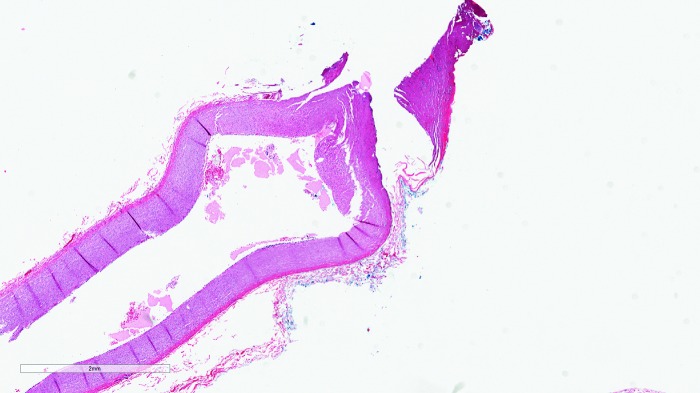
Seal quality and thermal spread on an artery using Ligasure—section of artery with moderate to sever cautery changes seen in the vessel wall with adherent fibrin and moderate loss of cellular detail of the vessel wall (*upper right*). The cautery changes permeate nearly through the vessel wall with associated mild thinning of the wall.

**FIG. 4.**
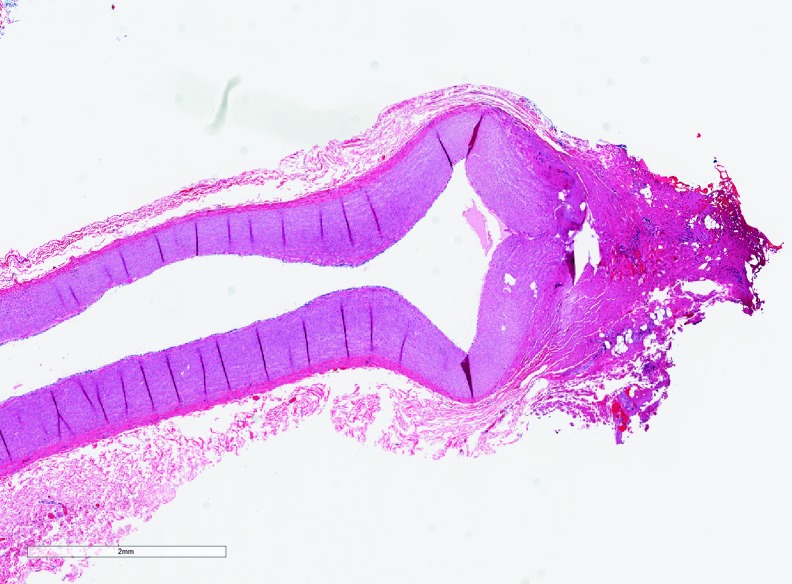
Seal quality and thermal spread on an artery using EnSeal—section of artery with moderate cautery changes seen in the vessel wall with mild loss of cellular detail of the vessel wall (*upper right*).

Thermal spread for each VSD is provided in Table 5. For arteries, C5, HA, HA7, LS, and ES had a mean thermal spread of 3.2, 3.2, 2.9, 3.8, and 4.2 mm, respectively (*p* = 0.721; no pairwise differences were significant). For veins, C5, HA, HA7, LS, and ES had a mean thermal spread of 2.1, 2.9, 2.3, 3.4, and 3.9, respectively (*p* = 0.281; no pairwise differences were significant). Example slides of arterial and vein seal quality for each VSD are shown in Figures 1–4.

**Table 5.** Thermal Spread Measurements

*Devices*	*C5*	*HA*	*HA7*	*LS*	*ES*	p	p*-Value for pairwise comparisons (Tukey adjustment for multiple comparisons)*
Arteries							
*N*	5	5	5	5	5		
Size, mm (mean)	4	4.6	3	3.3	3.5	0.275	None significant
Jaw temperature, °C (mean)	114	166	168	87	98.3	**<0.001**^a^	C5<HA(0.001); LS<C5(0.001); LS<LS(0.002); LS<HA7(0.001)
Thermal energy spread, mean (mm)	3.0	3.2	2.9	3.8	4.2	0.721	None significant
Veins							
*N*	5	5	5	5	5		
Size, mm (mean)	3.8	4.2	3.6	3.5	4	1.348	None significant
Jaw temperature, C (mean)	108	162	158	94	92	**<0.001**^a^	LS<HA(0.001); LS<HA7(0.001); C5<HA(0.04); C5<HA7(0.001)
Thermal energy spread, mm (mean)	2.1	2.9	2.3	3.4	3.9	0.281	None significant

^a^ Significant at *p* < 0.05.

## Discussion

Contemporary VSDs differ in instrument design and the type of energy they deploy in the act of sealing and transecting arteries and veins during laparoscopic procedures. Although early generations of ultrasonic VSDs were limited in use to 3 mm vessels, subsequent advances, including tissue feedback algorithms, have allowed contemporary technology to be relevant in addressing vessels up to 7 mm.^8,9^

The primary outcome of this study was to test vessel sealing quality of five contemporary VSDs measured by the maximum bursting pressure, which defines the efficacy of VSD.^8^ Specifically, we aimed to test the VSDs on small (2–5 mm), medium (5.1–7 mm), and large (>7 mm) arteries and veins. For the bursting pressure trials, C5, at both distal and proximal jaws, provided the highest bursting pressure measurements across all vessel sizes. For small and medium size vessels HA7, LS, and ES also demonstrated equivalent bursting pressures. However, all three VSDs demonstrated significantly lower bursting pressures when they were used for large size vessels. HA demonstrated the lowest bursting pressures for all three vessel sizes.

Bursting pressure failure was defined by threshold of 300 mm Hg for arteries and 30 mm Hg for veins.^8,10^ Again, C5, HA7, and LS were all able to effectively seal arteries and veins up to 9 mm with no failures. HA and ES performed slightly less favorably. For medium and large size arteries, HA had bursting failure of 20% and 40%, respectively. ES was significantly less efficient with small, medium, and large arteries with bursting failure rates of 10%, 40%, and 80%, respectively.

The quality of vessel sealing and amount of thermal spread are dependent on several factors, including vessel type, vessel size, temperature, time, and the type of energy used.^11,12^ In addition, adequate and even compression force throughout the entire length of the jaw is crucial for the creation of a stable and reliable seal.^10,13,14^ Uniform compression is very much related to the jaw configuration of the VSD. Most contemporary VSDs utilize either a scissor-like or pivoting jaw design. It has been shown that the scissor-like design is associated with a less uniform distribution of compressive forces with decreasing pressure force from the proximal to the distal end of the jaws, resulting in a weaker sealing quality at the tip of the instrument.^12,13^ C5 is designed with a novel pivoting jaw to address the limitations of the scissor-like jaw configuration. Specifically, the C5 device evaluated in this study consists of a novel pivoting jaw design and closing mechanism with the sealing electrodes distributed in both the upper and lower jaws.^14^ This mechanism allows for a more homogeneous pressure distribution in the entire jaws. Our data with the C5 support these earlier findings.

All VSDs should be used with great care adjacent to vital organs, and a margin of at least 5 mm is recommended to avoid any thermal damage.^5^ For both mesenteric and vessel tissues, ES and LS demonstrated the lowest jaw temperatures, whereas HA and HA7 consistently demonstrated the highest jaw temperatures across all vessel sizes. These data are consistent with previously reported studies.^5,15,16^ ES and LS employ a pulsed bipolar energy and a feedback energy control output during tissue sealing and transection, which maintain the jaw temperature levels below 100°C.^17^ However, of even greater importance than the recorded jaw temperature is the degree of coagulation necrosis on either side of the jaws. In this regard, HA and HA7 had the least thermal spread for both veins and arteries compared with the other three VSDs. It may seem counterintuitive that the devices with the highest jaw temperature resulted in the least energy damage on histopathology. However, it is clear that many factors affect tissue response. The advantage of high energy is because of a feedback loop that delivers fast and highly efficient energy with the least amount of transection time. Reyes and colleagues evaluated optimal temperature and duration of clamp time to achieve a complete vessel seal. Vessel seals tested at 40°C, 60°C, and 80°C demonstrated 25%, 17%, and 2.8% failure rates, respectively. There were no failures at 90°C applied for 10 seconds. These investigators concluded that average of 2.4–3.8 MPa (348–551 psi) pressure force with 90°C with at least 10 seconds of clamp time is required for optimal and reliable vessel sealing.^10^ Current prices of energy devices are HA7—$507, HA—$48, C5—$450, LS—$470, and ES—$487. These prices were taken from the Emergency Care Research Institute (ECRI) national supplies database that captures reported purchase price from hospitals across the country (prices assessed on October 12, 2017).

Finally, we evaluated tissue sticking, charring, and carbonization for all VSDs. Tissue sticking on the instrument's jaws can create increased resistance to energy transmission, thereby prolonging the sealing time and subsequent coagulation necrosis on either side of the jaws. C5, in both proximal and distal jaw positions, had the lowest tissue sticking scores followed by HA, ES, LS, and HA7 when used on small and medium vessels. However, for large arteries, ES and HA7 demonstrated best results followed by C5, LS, and HA. Our data are similar to results reported by Milsom and colleagues,^16^ in which they demonstrated that LS had poor tissue sticking results compared with ES and HA.

Overall, all VSDs created a reliable seal for small and medium size arteries. For large arteries, C5, HA7, and LS were the most reliable VSDs with the highest bursting pressures and no bursting failures <300 mm Hg. HA and HA7 are associated with highest jaw temperatures but both provide the fastest sealing and transection time with less thermal damage.

This study has several limitations. The main limitation is that the findings for VSDs in an animal model may not be similar in humans. However, previous comparison studies have shown that swine is the most optimal model for studying vessel anatomy and physiology. Another limitation is that all procedures were performed through an open rather than laparoscopic approach. The two environments are markedly different with respect to air flow and other parameters that might affect jaw temperature. In addition, the list of energy devices evaluated in this study is not exhaustive. There are multiple other devices that are routinely used in clinical practice in the United States. However, our goal was to test the most commonly used devices and compare them with a novel Caiman device. In addition, our sample size was limited to only 10 trials with each instrument. Finally, all vessels were evaluated by two surgeons who were not blinded and this may have introduced some bias to the study results.

## Conclusions

All tested VSDs provided excellent seal quality as corroborated by histopathology and supraphysiologic bursting pressures. C5 provided the highest bursting pressure measurements at both proximal and distal jaws and consistently scored better than the other devices with regard to tissue sticking, charring, and carbonization. However, HA and HA7 provided the least thermal damage, whereas LS and ES provided the lowest jaw temperatures, although with a broader area of thermal damage.

## Abbreviations Used

C5: Caiman 5
ES: EnSeal G2
HA: Harmonic Ace Plus
HA7: Harmonic Ace +7
LS: LigaSure (5 mm to 37 cm, blunt tip laparoscopic sealer)
VSDs: vessel sealing devices
